# Ex-Vivo Evaluation of “First Tip Closing” Radiofrequency Vessel Sealing Devices for Swine Small Intestinal Transection

**DOI:** 10.3390/vetsci9080445

**Published:** 2022-08-19

**Authors:** Luca Lacitignola, Alberto Crovace, Giuseppe Passantino, Francesco Staffieri

**Affiliations:** 1 Department of Emergencies and Organ Transplantation, Section of Veterinary Clinics and Animal Production, University of Bari, Valenzano, 70010 Bari, Italy; 2 Department of Veterinary, Medicine University of Bari, Valenzano, 70010 Bari, Italy

**Keywords:** jejunum, radiofrequency vessel sealing device, intestinal thermofusion, swine

## Abstract

**Simple Summary:**

The study observed the burst pressure (BP), number of activations, and histological evaluation of ex vivo swine small intestinal loops transected by stapler, a single fulcrum radiofrequency vessel sealing (RFVS—Ligasure Atlas) device, and the newly-invented jaws RFVS (Caiman). Caiman5, Caiman Maryland, Caiman12, Ligasure Atlas, and Stapler were employed as experimental groups, with Stapler serving as the control group. Caiman5, Caiman12, and the stapler needed just one activation to complete the seal. The Caiman5 and Caiman Maryland groups had considerably lower mean blood pressures than the Stapler group. The RFVS Caiman12 and Ligasure Atlas generated mean BP results that were comparable to the Stapler and did not vary. Caiman12 and Ligasure Atlas generate equivalent mechanical capabilities as well as stapled intestinal closure, while Caiman12 requires just one activation to complete the transection.

**Abstract:**

This study compared burst pressure (BP), number of activations, and histological assessment of ex vivo swine small intestine loops transected by stapler, a single fulcrum radiofrequency vessel sealing (RFVS) device, and the newly-developed jaws RFVS. Fifty (*n* = 50) 20 cm long jejunal loops were randomly assigned to be transected with RFVS devices and linear stapler (Caiman5, Caiman Maryland, Caiman12, Ligasure Atlas, and Stapler group as control respectively). Caiman5, Caiman12 and stapler required only one activation to complete the sealing. The mean BP in Caiman5 and Caiman Maryland groups were significantly lower (*p* < 0.05) than the S group as control and the other RFVS devices studied. RFVS Caiman12 and Ligasure Atlas produced mean BP values that were close to the Control and did not differ between them. The lumen was totally closed in the Caiman12 and Ligasure Atlas groups. The findings of this investigation were promising; we discovered that Caiman12 and Ligasure Atlas produce comparable mechanical capabilities as well as stapled intestinal closure, however Caiman12 need a single activation to complete the transection.

## 1. Introduction

Hand sewn sutures and staples remain the gold standard for intestinal stump closure and anastomosis. However the use of radiofrequency (RF)-based technologies were previously examined [1]. RF devices allow simultaneous sealing and cutting of structures similarly to staplers, but using a principle based on structure coagulation, by an energy delivery based on an impedance feedback reading system, in combination with mechanical pressure to cause a physical denaturation and reconfiguration of cellular proteins, sealing the structure extremities with minimal thermal spread injury. The advantage is to achieve effective and safe tissue resection quickly, technically less demanding (compared to hand sutures) and economical (avoiding the use of expensive stapler charges). Although the native use of this technology is aimed at blood vessels, several authors have investigated the use of similar technologies on other tissues such as the intestine. The studies in human patients and in animal models have shown that bipolar RF technology has been reported as an alternate approach for intestinal fusion and is used to seal intestines for transection [2,3,4,5,6,7] or anastomosis [2,8,9,10,11,12,13,14,15,16]. In a previous study, our research group founded that not all radiofrequency vessel-sealing (RFVS) devices performed small intestine transection at the same level in terms of bursting pressure. As a result, only a 10 mm width instruments showed similar burst pressure (BP) values to stapled closure [17]. However, the study showed that several coagulation cycles need to be performed to complete the intestinal closure, due to the length of instrument jaws and compression force.

A new RFVS instrument has just been released to the medical market. The fundamental characteristic of this instrument is the jaw design, which consists of lengthy jaws (ranging in length from 21.5 mm to 50 mm) with a double fulcrum that produces a “first tip closure” and a uniform compression force between jaws when closed.

We hypothesized that the innovative characteristics of this RFVS device will improve small intestinal loop closure performance when compared to prior examined devices.

As a consequence, the current study compared burst pressure, number of activations, and histological assessment of ex vivo swine small intestine loops transected by stapler, a single fulcrum 10 mm width RFVS device, and newly developed jaws of 12 mm, 5 mm, and Maryland RFVS.

## 2. Materials and Methods

### 2.1. Samples

Fifty (*n* = 50) 20 cm long jejunal samples (diameter 2 cm, thickness 3 mm) were collected from four healthy female Large White pigs weighing 60 kg and slaughtered at a nearby slaughterhouse. According to national legislation, no permission from the Ethical Committee was required because the specimens were collected from slain animals.

The samples were preserved in saline solution after slaughter and transferred in a refrigerated box (4 °C) to the Unit of Veterinary Clinics and Animal Production, University of Bari, Italy. The samples were then kept at room temperature for 60 min. Experiments were carried out 90 min following harvesting. The samples were randomly assigned to five separate groups of 10 each, using a randomization list downloaded from a website. (www.randomization.com) assessed on 10 January 2022.

### 2.2. Experimental Groups

**Figure 1:**
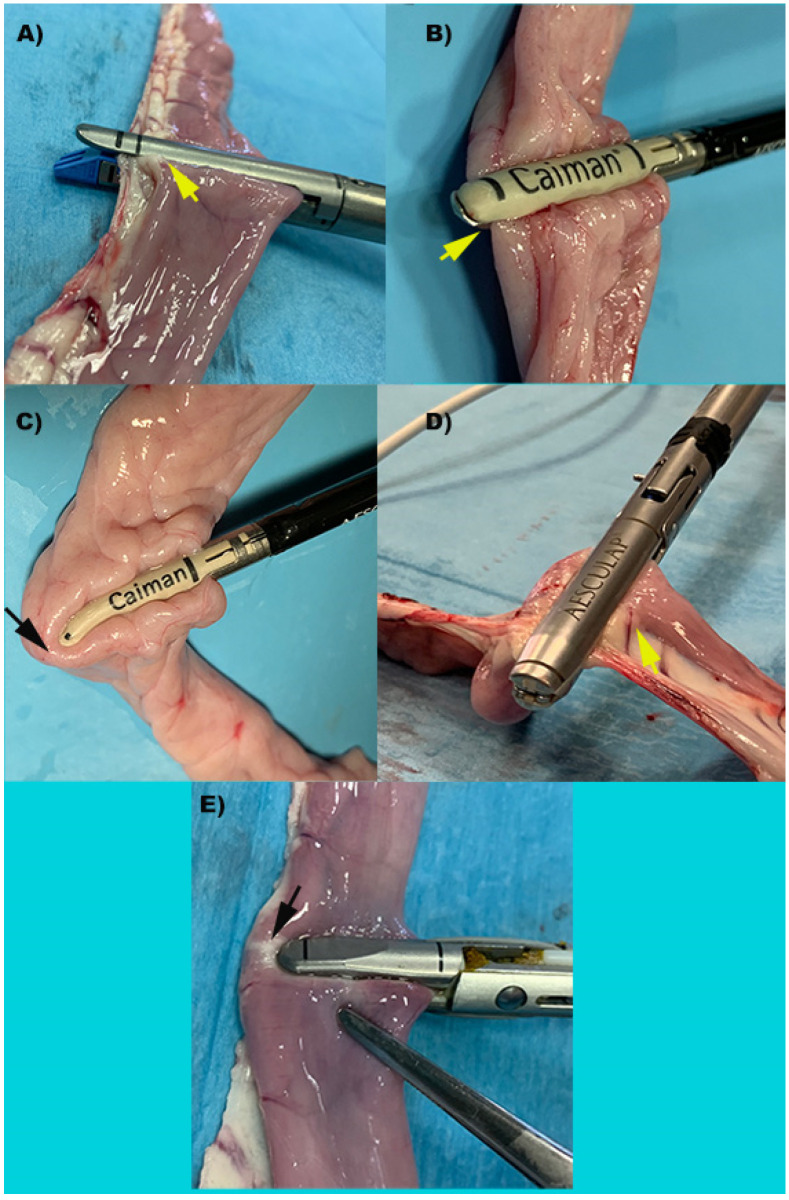
Representative images of intestinal loops during transection performed by each device. (**A**) S: Stapler, representing the control group; (**B**) Caiman5; (**C**) Caiman Maryland; (**D**) Caiman12; (**E**) Ligasure Atlas. Yellow arrows show the entire loop is included between the device’s jaws. Black arrows show that the device’s jaw is shorter than the loop diameter resulting in incomplete loop clamping with a single bite.

**Figure 2:**
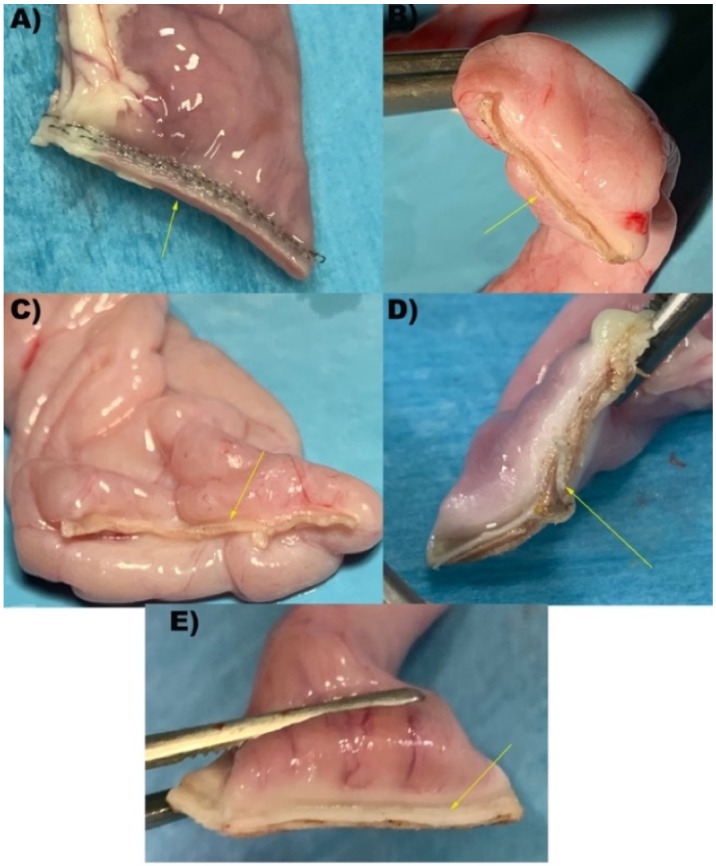
Representative images of intestinal loops after transection performed by each device. (**A**) S: Stapler, representing the control group; (**B**) Caiman5; (**C**) Caiman Maryland; (**D**) Caiman12; (**E**) Ligasure Atlas. Yellow arrows show the sealing line performed.

In group S (*n* = 10), a 45 mm endoscopic stapler (Endopath Ets 45 mm Articulating Linear Cutter, Ref. Ats45, Ethicon Endosurgery Inc., Cincinnati, OH, USA) was employed with blue cartridge 3.5 mm titanium staples (Endopath Ets45 3.5 mm, Ref. Tr45b, Ethicon Endosurgery Inc.). In the Caiman5 group (*n* = 10) the loops were closed and transected by a 5 mm wide, 26.5 mm long straight jaws (Caiman 5, Aesculap BBraun). In the Caiman Maryland group, the same procedure was performed by 5 mm wide 21.4 mm long Maryland jaws (Caiman Maryland, Aesculap BBraun). In the Caiman12 group loop closure was performed with 12 mm wide and 50 mm long straight jaws (Caiman 12, Aesculap BBraun). Handpieces used in the Caiman5, Caiman Maryland, Caiman12 groups were connected at the same generator (Caiman Lektrafuse RF Generator, Aesculap BBraun), and the power set at standard option. In the group Ligasure Atlas (*n* = 10) the transection was performed with a 10 mm radiofrequency vessel sealing device with straight, 21.4 mm long jaws (LigaSure Atlas Tissue Fusion Laparoscopic Instrument, 37 cm, ref LS1037, Medtronic, Milan, Italy). The instrument used in the Ligasure Atlas group was connected to a generator (ForceTriad, Medtronic) with a power setting at 3 bars. (Figure 1 and Figure 2). The number of activations and gross evaluation of sealing was recorded.

### 2.3. Sample Constructs & Burst Pressures

Each specimen then was sealed and transected using the previously randomly assigned method. Once the transection was performed, each specimen was stored in different boxes containing saline solution and stored at room temperature. Following that, each sample was prepared for burst pressure measurement using the previously stated procedure [17]. Briefly, each setup needed the connection of an infusion set line inside the jejunal loop lumen for air administration. The tube line was then tightened using a 4 mm polyethylene cable tie, taking care not to let air leak around the tube. A three-way stopcock was coupled to the air infusion line. To inflate the stopcock-connected construct, an air pump was employed. A digital manometer was attached to the stopcock’s other connector for continuous recording at maximum pressure. Maximum pressure in mmHg of the construct was recorded.

### 2.4. Histology

The transected tract obtained from the half tract of intestinal loops not intended for pressure testing were fixed in 10% neutral-buffered formalin, routinely processed, embedded in paraffin, cut at 3–5 µm, and stained with hematoxylin and eosin (H & E). Intestinal layer architecture, thermal and mechanical damage, compression, and complete lumen closure were evaluated.

### 2.5. Statistical Analysis

Statistical analysis was performed to compare the experimental groups versus the control group with available software (The jamovi project 2021; jamovi. Version 2.2). Retrieved from https://www.jamovi.org. assessed on 15 January 2022) Data were assessed for normality of distribution with the Kolmogorov–Smirnov test. Data were reported as the mean ± SD, and range. A one-way ANOVA was used to compare results among groups and *p*-values < 0.05 were considered significant. Power analysis was performed by a specific analysis software G*Power 3.1, Dusseldorf, DE, Statistical Power Analyses). A priori analysis for burst pressure (BP) variable showed power >80%.

## 3. Results

### 3.1. Number of Activations

To complete the loop transection, Stapler (control), Caiman5, and Caiman12 required just one activation. Caiman Maryland and Ligasure Atlas, on the other hand, resulted in a median of two activations for full loop cut and sealing.

### 3.2. Burst Pressure

**Figure 3:**
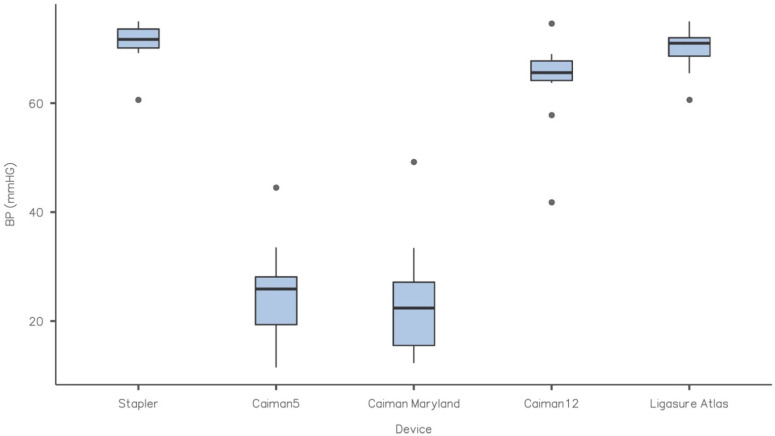
Box-plot of BP (mmHg) for each experimental group. Each box represents data from the 25th to the 75th percentiles, the line in the box represents the median, the whiskers represent the range. Dots are outliers. Caiman12 and Ligasure Atlas gave mean BP values that were similar to the control and did not differ (*p* > 0.05). The Caiman5 and Caiman Maryland had considerably lower mean BP values (*p* < 0.05) versus the control.

All specimens were transected with all RFVS devices, and no faults were detected at the sealing line. As a consequence, all samples were tested for BP. Table 1 and the figure summarize the results of the burst pressure tests. The Caiman5 and Caiman Maryland did not differ significantly (*p* > 0.05). The mean BP values, on the other hand, were significantly lower (*p* < 0.05) than the control and the other RFVS devices studied. Caiman12 and Ligasure Atlas, on the other hand, produced mean BP values that were close to the control and did not differ between them (*p* > 0.05) (Figure 3).

### 3.3. Hystology

**Figure 4:**
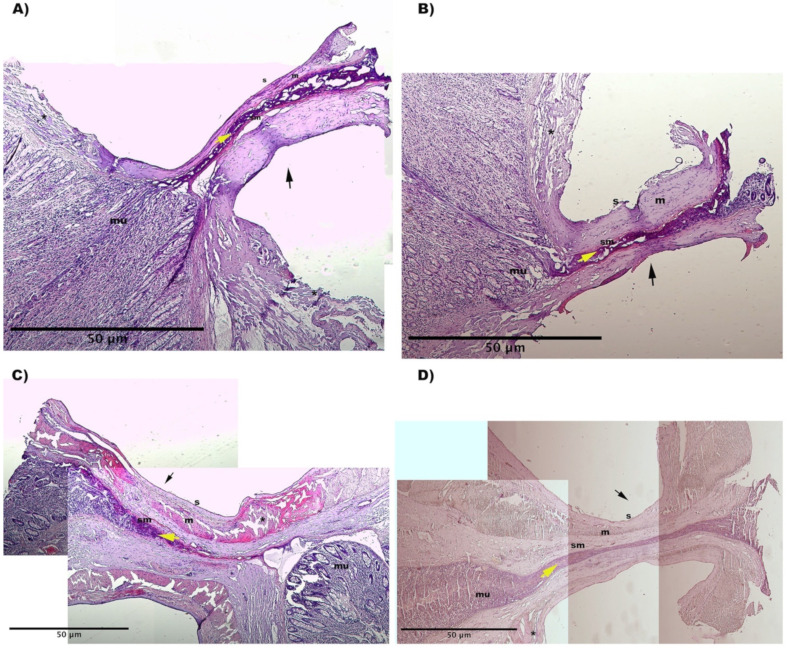
Representative histological images from: (**A**) Caiman5 group, (**B**) Caiman Maryland group, (**C**) Caiman12 group (**D**) Ligasure Atlas group). H&E stain. The images were obtained by combining several 4X magnification fields of view. Black arrow shows the fusion line. (**s**) serosa; (**m**) muscolaris; (**sm**) submucosa; (**mu**) mucosa. Yellow arrow shows the clot plug inside the sealed lumen. (*) thermal injury at sero-muscular layers.

The presence of titanium staples prohibited effective processing of the samples, hence analysis of samples closed with the stapler was not studied. Furthermore, we evaluated staple removal unsuitable for evaluating closure quality. Thermal and mechanical damage was found around the closure point of all RFVS devices specimens. The tissue seemed compressed and significantly elongated, with altered architecture. The mucosal architecture was destroyed, resulting in a thermal coagulum with holes in the tissue and no discernible cellular architecture. The lumen was totally closed in the Caiman12 and Ligasure Atlas groups, with the mucosal and muscle layers resting close to each other. The mucosal layer showed as a thin structure, indicating the efficacy of the compression used and the sealing success (Figure 4).

## 4. Discussion

Our hypothesis was partially proven. In reality, we examined numerous handpieces of varying form and diameter, but only the Caiman12 mm width device with the “first tip closure” and Ligasure Atlas produced BP values comparable to the control group and previously published RFVS device.

The results indicated that instruments with longer jaws (Caiman5, Caiman12, and Stapler) only completed one cycle every bite. The instruments employed in Caiman Maryland and Ligasure Atlas, on the other hand, required a median of two activations to complete the loop transection, due to shorter jaws. Although it seems logical that longer jaws may bite more tissue, the maximal BP has been characterized as being affected by the power level and the number of cycles administered at the same bite (without extending the jaws) [3]. Coagulation cycles that are repeated can potentially impact the healing process during in vivo operations. In fact, even though the thermal spread of the RFVS devices is fairly small, using many coagulation cycles increases the danger of delayed thermal injury, predisposing to intestinal leakage, total dehiscence, or failure of intestinal closure. In any event, the precise energy provided cannot be estimated since all of the RFVS devices used in this investigation featured a feedback-controlled energy adjustment that measured the tissue impedance beginning with the device closure during the sealing cycle. An algorithm within the generator altered the output based on the changes in tissue impedance based on these readings. Thus, the use of instruments with longer jaws might prevent duplicate or more activation to complete resection, preventing excessive heat impacts on tissues that would have a detrimental influence on the quality of sealing [3].

The compressive pressure applied by the instrument’s jaws has been shown also to have an effect on tissue sealing [2,16,18,19,20,21]. The ideal compression for the small bowel has been shown to be 0.15–0.25 MPa [1]. The compression pressure mechanisms of the RFVS devices examined in this study differed. The compression of the Ligasure Atlas, as well as the stapler, compressed the tissue at the base of the jaws and gradually reduced towards the tip [18,20]. Furthermore, compression pressure has been proven to be non-uniform along the jaws of the devices. On the contrary the Caiman5, Caiman Maryland, and Caiman12 have a double fulcrum on the lower branch that enable the tip closing first. This feature avoids the tissue slipping from the tip and provides significant uniform pressure along the jaws [20,22].

Maximum BP values in our research were 25.5 mmHg for Caiman5, 24.0 mmHg Caiman Maryland, 63.9 mmHg For Caiman12, 70.0 mmHg for Ligasure Atlas, and 71.1 mmHg for S as control. Maximum burst pressure was substantially lower (*p* < 0.05) in the Caiman5 and Caiman Maryland groups than in the Caiman12, Ligasure Atlas, and S (control) groups. In other reports, the highest BP stated ranged from 39.8 to 60.28 mmHg [2,3,8], however instruments with a diameter of 5 mm demonstrated the worst sealing. However, in fastened or fed pigs, small intestinal pressure were documented <75 mmHg [23], while in dog species, the physiological motility pressure recorded in vivo does not surpass 25 mmHg [24,25]. On the contrary, both 10 and 12 mm (Ligasure Atlas and Caiman12) width devices exceeded the physiological range by more than twofold and did not differ substantially from the stapler closure, which is considered the gold standard. As a result, we may guess that the mechanical features of Caiman12 and Ligasure Atlas may guarantee the same performance. However, we contend that the Caiman12 may cause less thermal injury due to its larger jaws and capacity to conduct a single uniform bite with only one coagulation cycle. The involvement of the healing process, on the other hand, should be thoroughly evaluated, which is a limitation of our study. In reality, the burst pressures for this study were obtained acutely after the transection, and the impact of healing could not be accounted for, therefore enhanced stability of the stump tissue could not be addressed. Based on established research, the sealing strength of the early phase increases dramatically with time [4,5]. In fact, anastomoses performed using RFVS devices were shown to remain intact [4], and increases BP when tested seven days post-operation [26]. Recently, in an animal model of thermofused anastomosis [27], anastomoses created by RFVS devices, set at different mode, were intact after two weeks in a proportion of 73.3–93.3 percent. Another study used an RFVS device to perform survivable anastomotic resection of the small bowel, resulting in intact seals with normal healing, 7 days after surgery [28].

The current study’s histology findings revealed that Caiman12 and Ligasure Atlas performed better in terms of seal and cut performance. We hypothesize that, in comparison to Caiman5 and 12, the complete closure of the lumen due to the formation of a firm and compact clot plug inside the lumen, as well as the uniform architecture obtained at the thermofusion lines, provided strengthening of the closed stump, resulting in higher BP values.

There are some limitations to this study. The ultimate clinical goal of this ex vivo animal model is to determine which RFVS device performs better for small intestinal sealing in order to be used during functional end to end stapled anastomosis (FEESA) or isoperistaltic side to side stapled anastomosis (ISSSA) in both open and minimally invasive procedures. However, while that Caiman12 and Ligasure Atlas yielded promising results providing comparable mechanical capabilities as well as stapled intestinal closure, complete anastomotic constructs should be studied to corroborate our findings.

We also evaluated the solely acute BP performances and hystological findings as limitations. Before expanding the use of RFVS intestinal sealing in a clinical situation in veterinary interest species, due to a lack of evidence about the exact role of the healing process post-operatively, the role of the healing process at various follow-ups must be examined. Thus, we can only speculate that Caiman12 and Ligasure Atlas could be limitedly used to prepare a temporary intestinal stump for end-to-end or side-to-side anastomosis (functional end to end stapled or isoperstaltic side to side anastomosis), enterectomies, or other procedures that require “sealing and cutting” an intestinal loop [8,29,30]. Furthermore, the current study’s findings are addressed to ex vivo swine intestine, and while this model is considered a translational model for human and small animal patients, particular clinical trials should be addressed before this technology is widely used.

## 5. Conclusions

In conclusion, we can speculate that Caiman12 and Ligasure Atlas are interesting candidates for additional research into small intestine RFVS anastomosis.

## Figures and Tables

**Table 1:** Burst Pressure (BP) in mmHg. Caiman12 and Ligasure Atlas gave mean BP values that were similar to the Control (S group) and did not differ (*p* > 0.05). The Caiman5 and Caiman Maryland had considerably lower mean BP values (*p* < 0.05) versus the control.

BP (mmHg)					
Device	Mean	Median	SD	Minimum	Maximum
**Stapler**	71.0	71.7	4.15	60.6	75.0
**Caiman5**	25.5	25.9	9.25	11.5	44.5
**Caiman Maryland**	24.0	22.4	11.25	12.3	49.2
**Caiman12**	63.9	65.6	8.82	41.8	74.6
**Ligasure Atlas**	70.0	71.0	4.27	60.6	75.0

## Data Availability

Data are contained within the article.
